# Development of ‘Lignin-First’ Approaches for the Valorization of Lignocellulosic Biomass

**DOI:** 10.3390/molecules25122815

**Published:** 2020-06-18

**Authors:** Tamás I. Korányi, Bálint Fridrich, Antonio Pineda, Katalin Barta

**Affiliations:** 1 Surface Chemistry and Catalysis Department, Centre for Energy Research, Konkoly Thege M. u. 29-33, 1121 Budapest, Hungary; 2 Stratingh Institute for Chemistry, University of Groningen, Nijenborgh 4, 9747 AG Groningen, The Netherlands; b.fridrich@rug.nl (B.F.); k.barta@rug.nl (K.B.); 3 Department of Organic Chemistry, University of Cordoba, Ed. Marie Curie (C 3), Campus of Rabanales, Ctra Nnal IV-A, Km 396, E14014 Cordoba, Spain; q82pipia@uco.es; 4 Department of Chemistry, Organic and Bioorganic Chemistry, University of Graz, Heinrichstrasse 28/II, 8010 Graz, Austria

**Keywords:** lignocellulose valorization, ‘lignin-first’, reductive catalytic fractionation

## Abstract

Currently, valorization of lignocellulosic biomass almost exclusively focuses on the production of pulp, paper, and bioethanol from its holocellulose constituent, while the remaining lignin part that comprises the highest carbon content, is burned and treated as waste. Lignin has a complex structure built up from propylphenolic subunits; therefore, its valorization to value-added products (aromatics, phenolics, biogasoline, etc.) is highly desirable. However, during the pulping processes, the original structure of native lignin changes to technical lignin. Due to this extensive structural modification, involving the cleavage of the β-O-4 moieties and the formation of recalcitrant C-C bonds, its catalytic depolymerization requires harsh reaction conditions. In order to apply mild conditions and to gain fewer and uniform products, a new strategy has emerged in the past few years, named ‘lignin-first’ or ‘reductive catalytic fractionation’ (RCF). This signifies lignin disassembly prior to carbohydrate valorization. The aim of the present work is to follow historically, year-by-year, the development of ‘lignin-first’ approach. A compact summary of reached achievements, future perspectives and remaining challenges is also given at the end of the review.

## 1. Introduction

Plant cells’ composite material is lignocellulose, which mainly consists of cellulose, hemicellulose, and lignin and in total accounts for ca. 90% of dry matter of land-based biomass. Depending on its origin, lignocellulose can be divided into three main categories—i.e., softwood, hardwood, and grass. In fact, the lignin content is highest in softwood, followed by hardwood and lowest in grasses [1,2].

**Figure 1:**
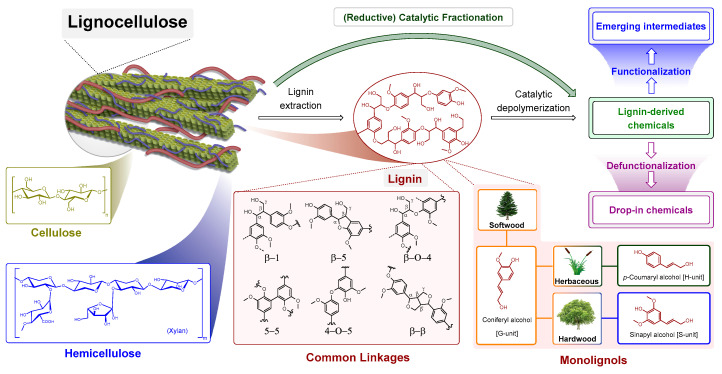
General strategies for lignocellulose valorization and application of lignin-derived platform chemicals with representative lignin structure displaying typical lignin subunits and linkages.

Cellulose and hemicellulose are both polysaccharides, differing in building units, degree of polymerization and morphology. Lignin is a complex aromatic biopolymer built up from three monolignols: p-coumaryl alcohol, coniferyl alcohol, sinapyl alcohol (Figure 1). These monolignols differ in the number of methoxy groups (none, one, and two) attached to the aromatic ring and make up the three key lignin units (H (hydroxyphenyl), G (guaiacyl), and S (syringyl), respectively). G units constitute approximately 90–95% of softwood lignin, whereas 25–50% of G and 50–75% of S units are typically found in hardwood lignin. Because the coupling of the monolignols is a process involving radicals, there are many possible linkages between the sub-units, involving different C-C and C-O bonds with certain linkages being more prevalent than others. A typical linkage in both softwood and hardwood lignins is the β-O-4 ether bond (Figure 1), approximately reaching half of the lignin in softwood and more than 60% in hardwood. Hardwood lignin contains less C-C linkages than softwood, because the additional methoxy groups on the aromatic rings, mainly in the S units, prevent their formation [1,2].

**Figure 2:**
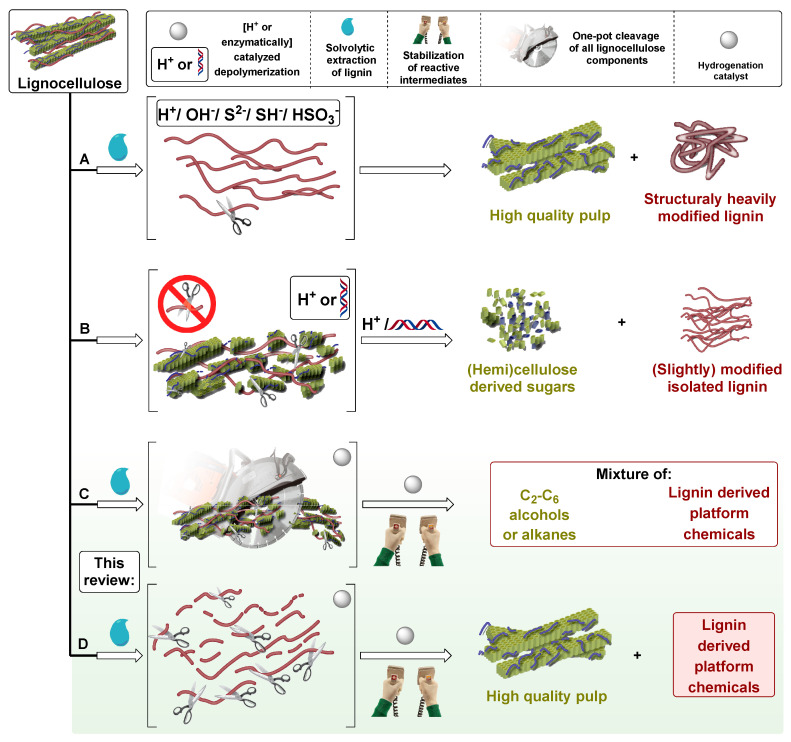
Types of lignocellulose valorization processes. (**A**) Harsh (pulping) fractionation. (**B**) Mild (enzymatic) fractionation. (**C**) One-step reductive catalytic processing. (**D**) Reductive catalytic fractionation (RCF).

Currently, only the cellulosic part of lignocellulosic biomass is used effectively as feedstock for the pulp and paper industry and as precursor of second-generation bioethanol. Traditionally, lignin is isolated from lignocellulosic biomass by fractionation in the pulp and paper industry (route ***a*** in Figure 2) and by fermentation in biorefineries producing cellulosic ethanol (route ***b*** in Figure 2). Pulping methods result in structurally heavily modified lignins (route ***a*** in Figure 2), while enzymatic lignin displays mild structural modification (route ***b*** in Figure 2) [3]. The chemical structure of native lignin is altered during conventional lignocellulose fractionation methods: ether bonds (β-O-4 and 4-O-5 in Figure 1) are cleaved and new stable C-C linkages are formed, resulting in more condensed and unreactive technical lignins [4]. Therefore, essentially under pulping conditions lignin undergoes structural rearrangement leading to the formation of unnatural C-C bonds [5]. High-yield lignin depolymerization methods are limited by the presence of these linkages formed during lignin extraction as well as the interunit carbon–carbon bonds within native lignin [6].

Several methods have investigated the catalytic conversion of recalcitrant lignins obtained from the pulp and paper industry, generally requiring harsher reaction conditions in order to achieve feasible product yields [2,3,5,7,8,9]. On the other hand, lignins obtained upon milder enzymatic digestion would lead to higher aromatic monomer yields, after catalytic depolymerization [1,10,11]. Many studies focusing on catalyst development for lignin depolymerization have used organosolv lignins prepared in the respective laboratories, using different fractionation procedures, which in terms of severity would fall between the industrial pulping conditions and enzymatic digestion [2,3,5,7]. Lignin valorization leads to value-added products such as biofuels, macromolecules (carbon fiber, polyurethane) and aromatics (BTX, monophenolic compounds) [12]. Functionalized lignin monomers can be regarded as perspective lignin-derived platform chemicals, from which emerging intermediates can be produced leading to pharmaceuticals, fine chemicals, polymers, and fuels. The other groups are drop-in chemicals with an existing market platform leading to bulk chemicals (Figure 1) [13].

Other approaches that emerged for the valorization of the whole lignocellulose are methods, which allow for the catalytic conversion of all constituents of lignocellulose, simultaneously. During these processes, product mixtures of cellulose as well as lignin are generated. For example, complete conversion of all lignocellulose components to lignin monomers and C_2_–C_6_ alcohols and/or alkanes is also possible by one-step reductive catalytic processing (route ***c*** in Figure 2) [3,13].

A method that emerged as alternative to lignin valorization is the so-called ‘lignin-first’ process. Already, several excellent reviews have discussed this powerful strategy [3,5,14,15,16,17,18,19,20]. Dubbed ’lignin-first’, this approach considers the catalytic conversion of lignin during biomass fractionation, in other words solvolytic extraction of lignin accompanied by instant lignin depolymerization and predominantly reductive stabilization of reactive intermediates (route ***d*** in Figure 2) [4]. This approach is also called ‘early-stage catalytic conversion of lignin’ (ECCL) [14] or, when using a metal catalyst under reductive atmosphere, as ’reductive catalytic fractionation’ (RCF) [15]. The extraction and immediate catalytic conversion of lignin to monomers by these methods from lignocellulose directly in the presence of a catalyst, usually under reductive conditions, results in higher yield of aromatic monomers due to the higher presence of cleavable C-O linkages and less C-C linkages [3]. RCF is also a two-step process, extracting lignin from whole biomass with a polar-protic solvent, and then selectively cleaving C-O ether bonds using a hydrogen donor and a heterogeneous catalyst. As a result, the process yields a depolymerized lignin oil rich in phenolic monomers, dimers, and oligomers, next to a solid carbohydrate pulp, which is amenable to further valorization. The most common solvents are alcohols (mainly methanol) and water/organic solvent mixtures such as water/dioxane and water/ethanol. The hydrogen donor can be pressurized hydrogen gas or can originate from the solvent or from the lignocellulose itself [16]. Generally, nickel or noble metal catalysts are used.

Different pathways and mechanisms have been proposed for the RCF process. The key steps in all cases are solvolysis as well as hydrogenolysis of ether bonds, removal of benzylic OH-groups (OH_α_), and possible removal of OH_γ_-groups. These primary reactions lead to the formation of substituted methoxyphenols and small oligomeric fragments. Additional hydrogenation of alkenyl and carbonyl groups, as well as hydrogenolysis, can take place (secondary reactions) [14,16,21].

**Figure 3:**
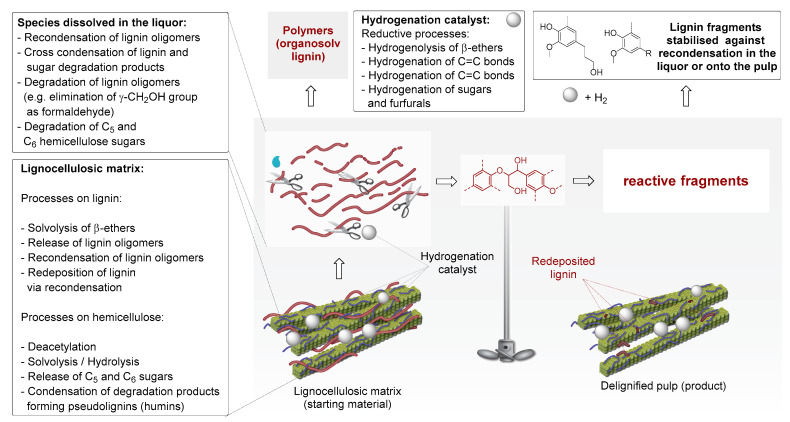
Schematic representation of chemical processes involved in the ‘lignin-first’ biorefining. For clarity, hemicellulose sugars and their degradation products were omitted.

**Figure 4:**
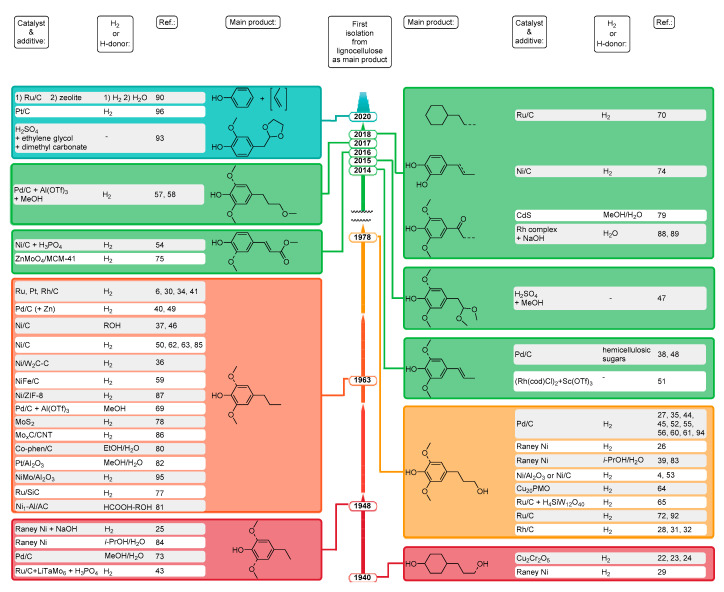
Main lignin monomeric products with functionalized sidechains, the date of their first isolation from lignocellulose as main product, typical catalysts, and additives with added hydrogen or hydrogen donors applied in the process, and literature references. For simplicity, guaiacols are not shown if the same syringol derivatives exist as main product. The detailed process characteristics and monomer yields of each individual study can be found in Table 1.

A detailed overview of RCF processes is presented in Figure 3 and discussed fully in the paper of Rinaldi et al. [21]. The lignin fragments present in the liquor are prone to several types of reaction. Under pulping conditions lignin fragments undergo recondensation, into technical lignins containing strong C-C bonds [14]. The main difference between pulping (route ***a*** in Figure 2) and these processes (routes ***c*** and ***d*** in Figure 2), is that in the latter two, catalytic processing of lignin happens in its native form in conjunction with fractionation, or parallel to cellulose processing. Under these conditions, reactive fragments or intermediates that originate during pulping immediately undergo stabilization (typically by hydrogenation see Figure 3) to form more stable molecules. Possible lignin-derived monomers obtained thus far from routes ***c*** and ***d*** combined (Figure 2) are collected in Figure 4.

The aim of this review is to follow historically the development of ‘lignin-first’ approach with inclusion of one-pot reductive catalytic depolymerization of all lignocellulosic components in the scientific literature. The number of these publications has increased significantly in the past years, new ideas emerged, and some novel methods were developed.

## 2. Chronological Overview

### 2.1. From 1940 to 2014

Originally, high pressure hydrogenation and hydrogenolysis of wood dates back to the 1940s with the aim of clarifying lignin structure [19]. Various woods (maple, aspen, spruce) were hydrogenated using copper-chromite, Raney-Ni, Pd/C, or Rh/C catalysts in dioxane(/water) solvent (mixture) under various conditions (173–280 °C, 35–333 bar H_2_, 5–20 h reaction time) resulted in 17–59 wt % monomer yields with 4-propylcyclohexanol (1940) [22], 4-ethylsyringol (1948) [25], 4-propylsyringol (1963) [26], 4-propanolguaiacol (1966) [27], and 4-propanolsyringol (1978) [30] as main products (entries 1–11 in Table 1 and Figure 4) [22,23,24,25,26,27,28,29,30,31,32]. Under harsh reaction conditions (250–280 °C, 240–333 bar H_2_ pressure) not only saturation of the benzene ring occurred, but also hydrogenation and hydrogenolysis of the holocellulose part took place [5,22,23,24]. The 4-n-propylphenol nature of the lignin monomers was confirmed in these early studies [5]. The efficiency of various (Ni, Pd, Rh, and Ru) catalysts was compared for the hydrogenolysis of spruce wood under mild reaction conditions (195 °C, 35 bar H_2_), the Pd/C catalyzed reaction gave the highest (16%) 4-propanolguaiacol yield, and the highest monomeric yield (34%) was obtained when Rh/C was used [5,28]. An increased monomers yield was demonstrated when softwood was replaced by hardwood [30], and hydrogen pressure did not affect the yield [31]. The increased monomers yield of hardwood can be explained by its higher S to G ratio compared to softwood, and therefore less C-C bonds, as was explained in the introduction.

Later, (1993–2011) new feedstocks (rice husks [33], birch [34], pine [35]) and catalysts (polyvalent metals [33], H_3_PO_4_ + Pt/C [34]) were tried in the dioxane(/water) solvent (mixture) under similar conditions and similar main products (4-propylsyringol and 4-propanolguaiacol) with comparable monomer yields (22–46 wt %) were gained (entries 12–14 in Table 1 and Figure 4). Four catalysts (Ru/C, Pd/C, Rh/C, and Pt/C) were tested for birch treatment and the monomer yields depended on the applied conditions: the total yield of monomers was 34% over the Pt/C catalyst, it was improved to 38% with addition of acid (H_3_PO_4_), the addition of dioxane further improved the yield to 46% [34]. The effect of additives was demonstrated in this work. Beside dimeric and oligomeric products, 4-propanolguaiacol was produced as a monomeric product almost exclusively in the Pd/C catalyzed hydrogenolysis of *Pinus radiata* wood [35]. Birch was converted in new solvents (ethylene glycol [36], methanol [37], ethanol/water mixture [38]) over new catalysts (Ni-W_2_C/C [36], Ni/C [37]) with high (47–54 wt %) monomer yields. Two processes [37,38] did not require addition of external hydrogen, alcohol solvents provided the active hydrogen species. One major monomeric product was generated in some processes (entries 12, 14, and 17 in Table 1) and a new one (4-propenylsyringol) appeared in 2014 (Figure 4) [33,35,38].

In 2012, Zhang and co-workers [36] described the direct conversion of birch over a Ni-W_2_C/C catalyst in a one-pot one-step reductive catalytic depolymerization process: the carbohydrate fraction was converted to ethylene glycol and other diols with a total yield of 76%, while the lignin component was transformed selectively into monophenols with a yield of 47% (entry 15 in Table 1 and 18% 4-propylsyringol in Figure 4). Different feedstocks, solvents and catalysts were tested. Hardwood, compared to softwood, led to a better conversion of both lignin and carbohydrates as expected. Replacing the original water solvent to methanol and later to ethylene glycol resulted increasing monophenol yields. Using a Pd/C catalyst led to the highest selectivity (56%) towards 4-propanolsyringol. This method uses high pressure (60 bar) hydrogen gas due to the complete conversion of all lignocellulose components (route ***c*** in Figure 2) [36].

In 2014, Ferrini and Rinaldi [39] realized that lignin was released solvolytically from the plant cell by simply “cooking” wood in the presence of Raney-Ni in 2-propanol (2-PrOH)/H_2_O) and partially depolymerized lignin, a non-pyrolytic lignin bio-oil was produced in addition to pulps that are amenable to enzymatic hydrolysis. The suspension of wood pellets, Raney Ni catalyst and aqueous solution of 2-propanol was heated under mechanical stirring (e.g., at 180 °C for 3 h) and 25 wt % lignin oil and 71 wt % pulp were produced (entry 18 in Table 1). The lignocellulosic feed was processed in the absence of molecular hydrogen and acetone generated by the hydrogen transfer can be hydrogenated to 2-PrOH. The holocellulose fraction or pulp (i.e., cellulose and hemicellulose) was isolated by filtration and washed with the 2-PrOH/water solution. Raney Ni was removed from the suspension with a magnet. Finally, the non-pyrolytic lignin bio-oil was isolated by solvent removal from the extracting liquor. This lignin oil is readily susceptible to further hydroprocessing (hydrodeoxygenation) under low-severity conditions. The complexity of the low-molecular weight lignin product mixture is a disadvantage, but the autogenous hydrogen usage and lignin-only conversion are advantages of this method. The pulp (holocellulose) is suitable for the production of glucose and xylose through enzymatic hydrolysis [39].

### 2.2. 2015

Lignin-first biorefineries were described in two works in 2015. Abu-Omar et al. [40] presented a selective hydrogenolysis of poplar wood with bimetallic Zn-Pd/C in methanol with external H_2_, focusing on the lignin monomers (30% 4-propylsyringol and 24% 4-propylguaiacol) and the enzymatic conversion of the retained pulp to glucose in 95% yield (entry 19 in Table 1). Sels et al. [41] presented reductive lignocellulose fractionation of birch sawdust through simultaneous solvolysis and catalytic hydrogenolysis in the presence of Ru/C in methanol under hydrogen at 250 °C resulting in carbohydrate pulp and lignin oil. The thermal and solvolytic disassembly of lignin (delignification) was immediately followed by the reductive stabilization of lignin’s most reactive intermediates into soluble and stable low-molecular-weight phenolic products. This fractionation strategy was denominated as a ‘lignin-first’ biorefinery, as the valorization of lignin to chemicals was performed before carbohydrate processing. The lignin oil yields above 50% of phenolic monomers (mainly 4-propylguaiacol and 4-propylsyringol) and about 20% of a set of phenolic dimers, relative to the original lignin content, next to phenolic oligomers. The separated carbohydrate pulp contains up to 92% of the initial polysaccharides (entry 20 in Table 1).

In a patent in 2015, Sels et al. described an interesting biorefinery concept for the direct production of light naphtha (hexane, pentane, methyl cyclopentane, cyclohexane, etc.) for converting lignocellulose in the presence of an acidic reactive aqueous phase and a redox catalyst (Ru/C + H_4_SiW_12_O_40_) in the organic extracting/reaction phase (entry 21 in Table 1) [42]. The products are useful as feedstock for steam and catalytic cracking, as precursors for the synthesis of bioaromatics, and as fuel additives. Another one-pot lignocellulose conversion into gasoline alkanes and monophenols was published by Ma et al. [43]. Raw biomass feedstocks (pine, corn, wheat, rice, etc.) were processed with Ru/C + LiTaMoO_6_ catalysts in phosphoric acid and pentanes and hexanes were produced from the carbohydrates with up to 82% total yield and monophenols, related alcohols and hydrocarbons from the lignin fraction. Partial hydrocracking of the monophenol fraction was suggested (entry 22 in Table 1).

Propylphenolics and propanolphenolics are the most abundant main lignin monomeric products using Ru/C + H_2_ and Pd/C + H_2_ catalytic systems, respectively, as shown in Figure 4. Accordingly, changing the catalyst from Ru/C to Pd/C drastically increased the OH-content of the phenolic monomers (entry 23 in Table 1) [44], and the solvent choice (methanol and ethylene glycol) has an impact on both pulp retention and delignification efficiency [45]. Reductive catalytic fractionation (RCF), as a new expression, was used first (2015) in the latter paper [45]. The effect of substrate and catalyst loading was studied over Ni/C catalyst in methanol: birch resulted higher monomer yields than poplar and eucalyptus, while higher catalysts loading caused higher monomer yields due to the presence of more hydrogen produced from methanol reforming [46].

As an example of metal-free catalytic systems (entry 26 in Table 1), the acid-catalyzed degradation of cedar and eucalyptus wood samples in a toluene-methanol solvent mixture resulted in selective production of lignin monomers, homovanillyl aldehyde dimethyl acetal and homosyringaldehyde dimethyl acetal (by 2015 in Figure 4), due to the trapping of enol intermediates with alcohol [47].

### 2.3. 2016

Luterbacher et al. reported that adding formaldehyde during biomass pretreatment followed by reductive depolymerization of this stabilized lignin over Ru/C as catalyst in methanol, produced guaiacyl and syringyl monomers at near theoretical yields (78 mol% for poplar) during hydrogenolysis (entry 27 in Table 1). These yields were three to seven times higher than those obtained without formaldehyde, which prevented lignin condensation by forming 1,3-dioxane structures with lignin side-chain hydroxyl groups [6].

In 2016, the utilization of hemicellulose as a hydrogen donor for the reductive lignin transformations and the separation of biomass into three main components: solid carbohydrate residue (mainly cellulose), liquid bio-oil (mainly lignin monomers and oligomers), and water-solubilized sugars (originating mainly from hemicellulose) emerged as a new idea [48]. Usage of Zn^II^ as a co-catalyst beside Pd/C increased the selectivity toward 4-propylsyringol and 4-propylguaiacol production (entry 29 in Table 1) through removal of the hydroxyl group at the Cγ position of the β-O-4 ether linkage [49]. All three major components of Miscanthus biomass (lignin, cellulose, and hemicellulose) were effectively (with 55% overall conversion) utilized into high value chemicals with mass balance of 98% using a Ni/C catalyst over 68% yield into four phenolic products from lignin (entry 30 in Table 1) [50].

Bruijnincx et al. described a tandem catalysis process for ether linkage cleavage within lignin, involving ether hydrolysis by water-tolerant Lewis acids (metal triflates) followed by aldehyde decarbonylation by a Rh complex (entry 31 in Table 1) [51]. In situ decarbonylation of the reactive aldehydes limited loss of monomers by recondensation, and surprisingly 4-(1-propenyl)phenols (4-propenylsyringol for poplar) were the main monomeric products (Figure 4). Hensen et al. also used metal triflate (Yb(OTf)_3_) catalysts for rapid cleavage of the chemical bonds between lignin and carbohydrates combined with Pd/C hydrogenolysis catalysts and 36–48% aromatic monomer yields (4-propanol derivatives, entry 32 in Table 1) were reached from different woods in the tandem process [52]. Xu et al. efficiently hydrogenated beech to natural phenolic alcohols (4-propanolsyringol and 4-propanolguaiacol) with 51% total yield using Ni/C catalyst in a methanol–water co-solvent (entry 33 in Table 1) [53]. Breaking the intramolecular hydrogen bonds in lignin β-O-4 motifs accelerated the C_β_-O cleavage, maintaining the original structure of lignin [53].

Román-Leshkov et al. investigated the RCF of corn stover using Ru/C and Ni/C catalysts and H_3_PO_4_ cocatalyst in methanol at 200 and 250 °C [54]. The monomer yields increased up to 38% as a function of time, with the addition of acid cocatalyst, and methyl coumarate/ferulate (2016 in Figure 4) were the main products (entry 34 in Table 1). Clear trade-offs existed between the levels of lignin extraction, monomer yields, and carbohydrate retention in the residual solids [54].

The influence of acidic and alkaline additives was studied on the Pd/C-catalyzed reductive processing of poplar wood in methanol: under acidic (H_3_PO_4_) conditions both delignification (to 4-propanolsyringol/guaiacol as main monomeric products, entry 35 in Table 1) and alcoholysis of hemicellulose are promoted, leaving behind a cellulose-rich pulp, alkaline (NaOH) conditions also enhanced delignification, but other lignin products (C_2_-substituted phenolics with loss of hydroxyl groups) were formed, lignin depolymerization was hampered, and cellulose loss was found in the pulp [55]. Synergetic effects of alcohol/water mixing were studied under similar conditions but without acid/alkaline addition in another paper. Low (30 vol %) water concentrations enhanced the removal of lignin from the biomass, while the majority of the carbohydrates were left untouched, high (70 vol %) water concentrations favored the solubilization of both hemicellulose and lignin, resulting in a cellulosic residue of higher purity [56].

### 2.4. 2017

The tandem metal triflate and Pd/C catalysis was further investigated by the Hensen group in 2017. Metal triflates were involved in cleaving not only ester and ether linkages between lignin and the carbohydrates, but also β-O-4 ether linkages within the aromatic lignin structure. Pd/C is required for cleaving α-O-4, 4-O-5 and β–β linkages. Synergy was revealed between Pd/C and metal triflates: under optimized conditions, 55 wt % mono-aromatics (entry 37 in Table 1)—mainly alkylmethoxyphenols (2017 in Figure 4)—were obtained from the lignin fraction (24 wt %) of birch wood [57]. Instead of metal triflates the effect of possible alternative acid co-catalysts (HCl, H_2_SO_4_, H_3_PO_4_, and CH_3_COOH) was studied for the tandem RCF process in another publication [58]. Al(OTf)_3_ and HCl, respectively, afforded 46 wt % (entry 38 in Table 1) and 44 wt % lignin monomers from oak wood sawdust in tandem catalytic systems with Pd/C at 180 °C in 2 h, therefore HCl is a promising alternative to the metal triflates [58].

Birch was effectively depolymerized using Ni-Fe/C catalyst with alloy structure in methanol reaching 40% monomer yield (entry 39 in Table 1) with 88% selectivity to 4-propylsyringol and 4-propylguaiacol [59]. *Pinus radiata* wood was depolymerized by mild hydrogenolysis in dioxane-water mixture by Pd/C catalyst to give an oil product, from which new biobased epoxy resins were prepared [60].

The role of Ni/Al_2_O_3_ catalyst was elucidated in the solubilization, depolymerization, and stabilization of lignin from birch in methanol in the 2017 work of Sels et al. [4]: the solvent is responsible for the first two processes, while the catalyst is for the stabilization through hydrogenation of reactive intermediates. Recuperation and reuse of the Ni/Al_2_O_3_ pellets was facilitated using a catalyst basket. This catalytic reduction also prevents undesirable repolymerization reactions.

**Figure 5:**
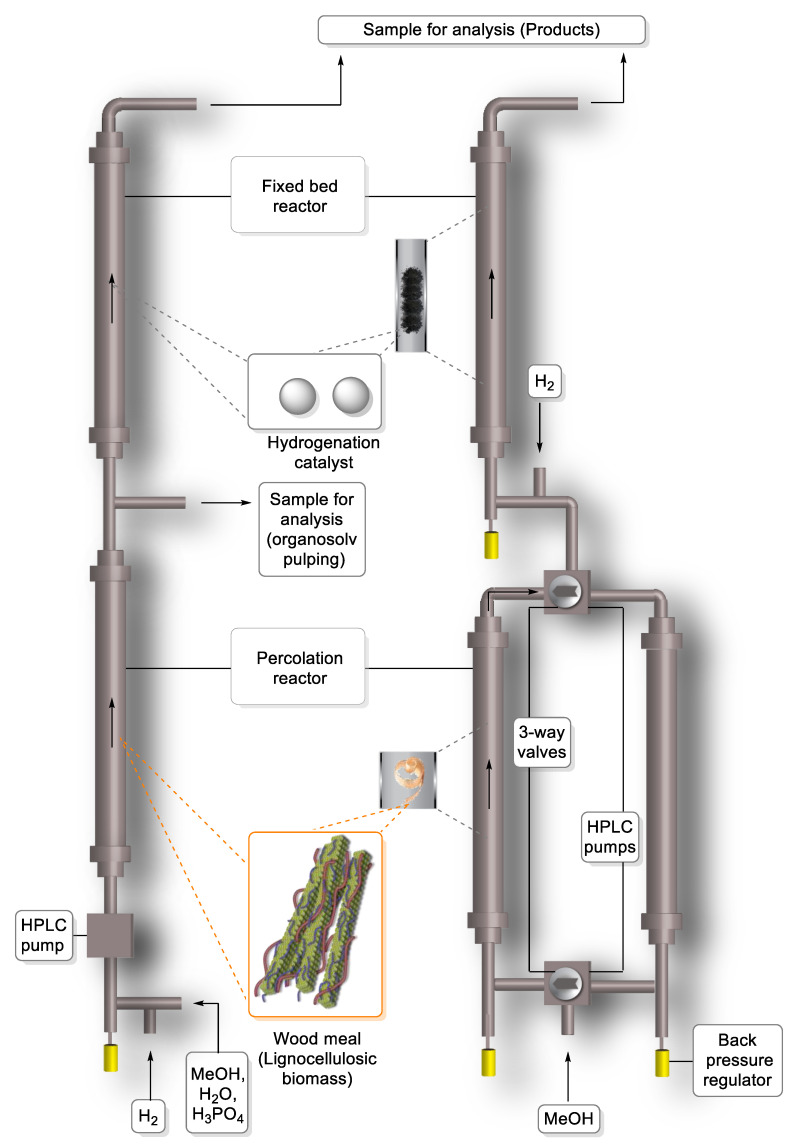
Schematic representations of semi-continuous flow-through reactor systems. (**Left**) Combination of a percolation reactor filled with woody biomass and a fixed catalytic bed reactor filled with Pd/C catalyst (Adapted from [61]). (**Right**) Schematic of the semi-continuous dual-bed flow reactor. Biomass in lower beds, catalyst in upper bed. (Adapted from [62,63]).

Continuous systems are vital for realistic scale-up because time-resolved product distributions and yields can be obtained from these experiments. The first two papers using flow-through systems instead of batch reactors for RCF were published by Samec [61] and Román-Leshkov [62] in 2017. During RCF in a flow-through system, separate reactors are used for pulping and delignification processes (Figure 5). A percolation reactor filled with birch and consecutively a fixed catalytic bed reactor filled with Pd/C catalyst was used in the first paper [61]. A methanol–water solution of phosphoric acid was percolated through the system at 30 bar pressure (left reactor system in Figure 5); under optimized conditions 37% yield of monophenolic compounds (18% propanolsyringol and 11% propylsyringol) was reached (entry 42 in Table 1). It was concluded that organosolv pulping and transfer hydrogenolysis should be performed under different conditions; and depolymerized lignin can be obtained without the palladium catalyzed step [61]. Two flow-through systems were used in the second paper [62]: a single-bed reactor with a biomass bed located upstream from a catalyst bed and a dual-bed reactor featuring switchable biomass beds physically separated from the catalyst in a separate upstream reactor (right reactor system in Figure 5). RCF of poplar with Ni/C catalyst in methanol solvent was studied in the latter paper, 17% monomer yield (mainly propylguaiacol and propylsyringol) was reached (entry 43 in Table 1). It was concluded that flow-through studies allowed the observation of biomass extraction intermediates, decoupling of solvolysis and hydrogenolysis, simple catalyst recovery and recyclability, and elucidated catalyst deactivation mechanisms [62].

### 2.5. 2018

Continuous systems initiated by Román-Leshkov et al. [62] were developed further in 2018 [63]. Kinetic studies of RCF in flow-through reactors revealed decoupling of the two limiting mechanistic steps, lignin solvolysis and reduction, which can be independently controlled. The difference of activation barriers between flow and batch reactors indicated that lignin extraction under typical RCF conditions was mass-transfer limited [63].

Complete lignocellulose conversion yielding valuable aromatics and fuels was introduced by Barta et al. [64]. In the first, mild depolymerization step following the principles of RCF using Cu_20_PMO (porous metal oxide), propanolguaiacol was obtained as main monomeric product (entry 45 in Table 1) that could be converted to plethora of value-added aromatic building blocks, also including amines. In the second step, the cellulose and unreacted lignin residues that were mixed with the heterogeneous catalyst were converted in supercritical methanol, resulting in aliphatic alcohols. Hydrothermal conditions suitable for full conversion of the residues allows for efficient catalyst recycling. Value-added products were produced in further catalytic pathways.

A comprehensive strategy for the smooth integration of an RCF-based biorefinery process into current petrorefinery schemes was carried out by Sels and coworkers [65]. Birch wood processed by RCF provided nearly theoretical amounts of phenolic monomers (entry 46 in Table 1 and Figure 4) and a solid carbohydrate pulp with 83% C_5_ and 93% C_6_ sugar retention in the presence of Ni/Al_2_O_3_ using methanol solvent [4]. This pulp can be converted either into bioethanol by fermentation [4] or to alkanes using liquid phase cellulose-to-naphtha (LPCtoN) technology with petrol as the organic solvent [65]. Bio-enriched gasoline was produced from the (hemi)cellulose pulp using a two-phase (water/fossil naphtha) catalytic slurry process followed by isomerization [65].

Rinaldi et al. [66] introduced a deep converting ‘lignin-first’ biorefinery concept in 2018, which means production of gasoline and kerosene/diesel drop-in fuels in two steps. Poplar and spruce were deconstructed over Raney Ni catalyst in isopropanol–water solvent mixture yielding lignin oils along with cellulosic pulps in the first step, next the lignin oils were catalytically upgraded to aliphatics or aromatics by simply changing hydrogen pressure and temperature in the presence of a Ni_2_P/SiO_2_ catalyst (entry 47 in Table 1). The self-sufficiency in hydrogen was achieved through the gasification of the delignified holocellulose. The role of Raney-Ni catalyst in the process was clarified in another paper by the Rinaldi group: it suppresses formic acid formation via sugar hydrogenation [67].

Alternatively, the use of hydrogen could also be avoided through the utilization of Fenton’s reagent (Fe^3+^, H_2_O_2_), that combined with enzymatic hydrolysis transformed sweet sorghum bagasse into phenolic monomers and sugars [68]. Initially, the feedstock’s molecular structure was modified through iron chelation and free radical oxidation via Fenton’s reagent. The lignin component of the modified feedstock was then selectively depolymerized in supercritical ethanol (250 °C, 6.5 MPa) under nitrogen to produce a phenolic oil (entry 49 in Table 1). Thus, Fenton’s reagent seems to provide beneficial effect in lignin depolymerization through the modification of lignin structure by hydroxylation and demethoxylation reactions of lignin substituents in the aromatic rings as well as by the formation of an iron-lignin complex. These two modifications are considered to make the ß-O-4 bond cleavage energetically more favorable. Fenton modification not only increased the yields of phenolic monomers, particularly ethyl-p-coumarate and ethyl-ferulate, but also enhanced enzymatic hydrolysis.

The metal triflate and Pd/C catalyst system was developed further by the Hensen group in 2018 [69]. Beside Al(OTf)_3_ other homogeneous acid catalysts were tried in the first fractionation step of a two-step process, where oak was converted to lignin-oil and cellulose pulp in the first step, then to phenolic monomers with up to 25 wt % yield over the Pd/C catalysts in the second step (entry 50 in Table 1). Phosphoric acid proved to be the most suitable catalyst because it minimized the repolymerization back to lignin in the first step.

A complete transformation of lignocellulose into valuable platform chemicals was reached by Wang et al. [70,71]. They transferred cornstalk into liquid alkylcyclohexanes (2018 1^st^ row in Figure 4, from the lignin fraction) and polyols (from cellulose and hemicellulose components) over Ru/C catalysts in aqueous phase in one step (entry 51 in Table 1) [70], and various biomasses (birch, beech, cornstalk, and pine) over Pd/C + Yb(OTf)_3_ catalysts in methanol solvent to bio-oil and carbohydrates in the first step (entry 52 in Table 1), then the lignin-oil to arenes over a Ru/Nb_2_O_5_ catalyst in isopropanol, and the carbohydrates phase to 5-hydroxymethylfurfural, and furfural in tetrahydrofuran/seawater in the subsequent steps [71] in high overall yields (entries 51 and 52 in Table 1).

Another RCF process was also published by the Sels group in 2018 [72]. They converted eucalyptus into lignin-derived (mono)phenolics, hemicellulose-derived polyols, and a cellulose pulp in butanol–water 1:1 mixture over Ru/C catalysts and 30 bar hydrogen with 49 wt % lignin monomer yields with propanolsyringol and propanolguaiacol as main lignin products (entry 53 in Table 1). Phase separation of n-butanol and water upon cooling offered a facile and effective strategy to isolate lignin-derived phenolics (n-butanol phase) from polyols (aqueous phase).

Carbohydrates served as an inherent hydrogen donor in a bark RCF process producing hydrocarbon bio-oil in gasoline and diesel ranges and 4-ethylguaiacol [73]. RCF of vanilla seeds was used to investigate the depolymerization of naturally occurring C-lignin, which consists solely of caffeyl alcohol units; only two products (propyl- and propenyl catechol, 2018 second row in Figure 4) were gained with 21 wt % lignin monomer yield (entry 55 in Table 1) [74]. Selective fragmentation into hydroxycinnamic esters (methyl coumarate and methyl ferulate (Figure 4)) was reached by RCF of corncob using MCM-41 supported ZnMoO_4_ catalyst in methanol [75]. The effect of support, added base, and solvent was studied in the catalytic depolymerization of cork over Rh/C catalyst: the highest bio-oil yield (43 wt %) was reached by a 2-methyl tetrahydrofuran/water ‘green’ solvent mixture (entry 57 in Table 1) [76]. The reusability of Ru/SiC compared to Ru/C catalyst was emphasized in the RCF of apple wood to lignin-oil further converted to jet fuel aromatics and polyurethane [77]. Unsupported MoS_2_ catalyst was used in the RCF of corn stover and 18 wt % phenolic monomers yield was reached (entry 59 in Table 1) [78].

Full utilization of biomass by means of solar energy was reached in 2018 by CdS quantum dots catalyzed cleavage of the β-O-4 bonds in birch into functionalized aromatics (2018 third row in Figure 4), xylose, and glucose under visible light at room temperature. The β-O-4 bond in lignin is cleaved by an electron–hole coupled photoredox mechanism based on a C_α_ radical intermediate, in which both photogenerated electrons and holes participate in the reaction. Due to the colloidal character of the catalyst it could be easily separated and recycled [79].

### 2.6. 2019

A new ‘lignin-first’ paper in 2019 discusses RCF of birch wood with high (up to 34 wt %) yields to monophenolic compounds (10% propylsyringol and 9% propenylsyringol) using Co-phen/C catalyst and formic acid or formate as a hydrogen donor (entry 61 in Table 1). The high yield was explained by transfer hydrogenolysis reactions of lignin fragments targeting the β-O-4′ bond and stabilizing reactive intermediates due to the cobalt catalyst [80]. Formic acid was also used as hydrogen source and as co-catalysts beside Ni-Al/C in another paper and a positive correlation was suggested between spillover hydrogen on the catalysts and lignin-derived phenolic monomer yields [81]. Pt/Al_2_O_3_ not only converted birch into phenolic monomers but also catalyzed methanol reforming in methanol-water mixtures to supply hydrogen. Increased lignin monomer yield (49 wt %, entry 63 in Table 1) was attributed to the stabilization of reactive lignin intermediates by hydrogenation of reactive bonds due to the higher hydrogen yield [82].

A proof-of-concept membrane filtration was demonstrated by Rinaldi and co-workers in 2019, for the separation and concentration of the monophenol-rich fraction (entry 64 in Table 1) from the lignin liquors (poplar, Raney Ni catalyst, isopropanol/water solvent/H-donor mixture) [83]. In a further paper, the impact of process severity (reaction temperature) was studied by the same group, using the same feedstock, catalysts, and solvent mixture in the second one [84]. Higher process temperatures led to improving overall delignification yields (up to 87%), producing low molar mass fragments and preferential cleavage of hydroxyl groups in monolignol sidechains via hydrodeoxygenation, yielding oils with lower oxygen content [84].

Ni/C-catalyzed delignification of poplar resulted recalcitrance to enzymatic digestion of cellulose. Subsequent gelatinization in trifluoroacetic acid greatly enhanced rates of enzymatic digestion or maleic acid-AlCl_3_ catalyzed conversion to hydroxymethylfurfural (HMF) and levulinic acid (LA). These results informed a ’no carbon left behind’ strategy to convert total woody biomass into lignin, cellulose, and hemicellulose value streams for the future biorefinery [85].

Two task-specific catalysts were developed for RCF: Mo_x_C/CNT for hardwood, and Ru/CMK-3 for softwood and grass. Using Mo_x_C/CNT for apple wood led to a carbohydrate (both cellulose and hemicellulose) retention degree in solid product close to theoretical maximum and a delignification degree as high as 98.1% with 42% lignin monomers yield (entry 67 in Table 1) [86].

Chemodivergent hydrogenolysis of eucalyptus sawdust was carried out with Ni@ZIF-8 catalyst: phenolic compounds having either a propyl or propanol end-chain were produced under different reaction conditions. Propanol-substituted phenols (10% propanolsyringol, 5% propanolguaiacol) at 220 °C and 30 bar H_2_ within 4 h were identified as the major depolymerized products, while propyl-substituted phenols (24% propylsyringol and propylguaiacol) where those at 260 °C and 30 bar H_2_ within 8h (entry 68 in Table 1) [87].

A homogeneous catalytic system (binuclear Rh complex) for a “lignin-first” biorefinery in water was applied for basswood producing aromatic ketones (2% propanonesyringol, and 1.6% ethanonesyringol (Figure 4)) with almost complete deconstruction of lignin component under mild conditions (110 °C, 1 bar Ar, 24 h, entry 69 in Table 1) [88].

### 2.7. 2020 

The same rhodium terpyridine complexes as homogeneous catalysts were used by the same group as in 2019 [88] for redox-neutral depolymerization of poplar wood under mild conditions affording aromatic ketones as the major monomer products (entry 70 in Table 1) [89].

The integrated biorefinery concept originated from Sels et al. [42,65] was developed further by the same group in 2020 [90]. The process model integrated three catalytic steps: RCF of wood, hydroprocessing of crude monomers extract, and dealkylation of crude alkylphenol product stream. Ru/C catalyst in methanol solvent converted birch in the first RCF step into a carbohydrate pulp amenable to bioethanol production and a lignin oil. Lignin monomers were cost-efficiently extracted from lignin oil with fossil n-hexane and were catalytically funneled into phenol and propylene (entry 71 in Table 1, and 2020, first row in Figure 4). A 78 wt % measure of birch was converted into xylochemicals [90]. An integrated techno-economic assessment of the biorefinery process that directly integrates the results of lab studies with economic costs and benefits was also developed [101]. They found that the scale of the plant, the feedstock-specific output quantities, and output prices highly determine the economic feasibility. The Sels group patented the chemocatalytic biorefinery concept [91]. Accordingly, three separate product fractions are produced in this biorefinery: (i) a lignin oil enriched with high contents of lignin-derived (mono)phenolics, (ii) essentially humin (furanic oligomers)-free hemicellulose-derived polyols, and (iii) a cellulose pulp. An example for eucalyptus sawdust is given as Entry 72 in Table 1.

Softwood lignocellulose was effectively (77–98%) depolymerized in a mild lignin-first acidolysis process (140 °C, 40 min, entry 73 in Table 1) using dimethyl carbonate and ethylene glycol solvents/stabilization agent producing high yield (9 wt %) of aromatic monophenols (2020, second row in Figure 4) and preserving cellulose as evidenced by a 85% glucose yield after enzymatic digestion [92]. The total utilization of lignin and carbohydrates in eucalyptus towards phenolics, levulinic acid, and furfural was emphasized in another paper in 2020: Pd/C catalyst in methanol solvent was used in the hydrogenolysis step, 50 wt % maximum phenolic monomers yield was achieved (entry 74 in Table 1) [93]. Beech wood was directly converted into lignin derived monomers (20 wt % yield) and dimers and holocellulose derived light hydrocarbons in the presence of a sulfided NiMo/Al_2_O_3_ catalyst in ethanol solvent at 260 °C (entry 75 in Table 1) [94].

Phenol was produced in a three-step process from pinewood with a 10 mol % overall yield [95]. In the first step pinewood was transformed into monomeric alkylmethoxyphenols using Pt/C catalyst in a methanol/water mixture as solvent at 230 °C and 30 bar H_2_ pressure by the selective cleavage of β-O-4 lignin bonds (entry 76 in Table 1). Subsequently, the methoxy groups were removed by combination of MoP/SiO_2_ and H-ZSM-5 catalysts leading to the formation of 4-alkylphenols—including 4-propylguaiacol, ethylguaiacol, and methylguaiacol—that were eventually dealkylated to phenol using H-ZSM-5 catalyst in the third step.

Zeolite-assisted fractionation of lignocellulose by preventing the recondensation reactions of aldehydes and allylic alcohols was achieved by Samec, Corma, and coworkers [96]. This prevention effect was attributed to the shape/size selectivity of protonic Beta zeolites, whose pore size limits undesired side reactions such as bimolecular condensations. In addition, mechanistic studies have pointed out that the reductive dehydration of allylic alcohols is carried out in the pores of metal-free zeolites. The highest lignin monomers yield from the organosolv pulping of birch wood was 20 wt %, using an ethanol/water mixture at 220 °C for two hours (entry 77 in Table 1). In parallel, furfural and ethylfurfural have been obtained as result of cellulose and hemicellulose fractions depolymerization over zeolitic acid sites.

Based on their previous kinetic studies of RCF [62,63], Román-Leshkov et al. [97] developed detailed mesoscale reaction-diffusion models for lignin-first fractionation. The models predict that mass transfer plays a governing role in solvolytic lignin extraction at the mesoscale. Lignin fragment diffusion competes with mass transfer resistance, which seems to be dominant effect when biomass particle size is over 2 nm. It is advisable to perform such tests for catalysts evaluation when the particle size of biomass is smaller than 2 nm, when the kinetics of the reaction is controlled by the diffusion of lignin fractions.

New concepts for lignin-first processes were introduced in 2020. Lignin-first integrated hydrothermal treatment [98], using an ionic liquid for lignin-first fractionation [99], and fractionation of wood with a γ-valerolactone consisting solvent system [100], was suggested. We do not consider the first two procedures as strictly regarded lignin-first processes as either a catalyst was not used [98], or reductive conditions were not applied [98,99]. The last procedure is remarkable, as a continuous operation was used to depolymerize maple wood lignin in a stirred reactor, which means continuous feeding of thermally pretreated lignin solution into the reactor and the products were collected at the outlet in a sample vial at 30 min intervals [100].

## 3. Summary

Early studies (1940–1986) on wood digestion or conversion using a catalyst were mainly focused on the elucidation of the chemical structure of lignin. The 4-n-propylphenol nature of the lignin monomers was confirmed [5]. With environmental concerns and increasing need to shift away from the dependence on fossil resources, interest in biomass as a sustainable feedstock has been resurgent, and lignocellulose has been identified as an important feedstock because it does not compete with the food supply. Innovative approaches, mainly related to efficient valorization of the cellulose platform have been introduced, together with the definition of top value-added platform chemicals. However, research in lignin has lagged behind due to its recalcitrant structure and it was only predominantly after 2010 that lignin conversion gained momentum and many fundamental works have been published, wherein significant progress has been made.

Notable advances in the one-pot full conversion of lignocellulose include the use of supercritical methanol as hydrogen source for the highly efficient conversion of lignocellulose to aliphatic small molecules in 2010 and 2011. The importance of supressing char formation has been recognized here. Later, interesting works to provide highly useful molecules in a one-pot, one-step process were the direct production of light naphtha (n-hexane, n-pentane, cyclohexane, and methylcyclopentane) by the Sels group in 2015 [42], which was developed further in 2018 [65] and 2020 [83] producing phenol and propylene. Liquid alkylcyclohexanes and polyols over Ru/C catalysts [70], and propylphenols, C_5_–C_6_ ketones, and furans over sulfided NiMo/Al_2_O_3_ catalysts [94] were formed in other one-pot one-step processes.

The concept of stabilization of reactive intermediates emerged in many different aspects in the field. One of the elegant examples of stabilization was introduced by Luterbacher et al. who described the role of formaldehyde and other carbonyl compounds in the protection of the native β-O-4 moiety during extraction, and thereby achieved much higher yields of desired monoaromatic products [6]. Trapping reactive intermediates originating from acid treatment after depolymerization in the form of acetals has also resulted in the suppression of recondensation and increased monomer yield from lignin. This approach has shown success on lignocellulose both in toluene/methanol mixtures [47], or more recently in dimethyl carbonate as solvent [84].

Undoubtedly, reductive catalytic fractionation (RCF) introduced during 2014–2015 by three research groups [39,40,41], has emerged as highly efficient method (also relying on stabilization) for lignocellulose valorization. Much research has been done regarding catalyst development, the role of additives, and types of hydrogen donors as well as reaction setup. For example using Zn-Pd/C bimetallic and Ru/C catalysts resulted in higher than 50% lignin monomers yield [40,41], isopropanol solvent ensured the source of hydrogen [39], and the direct production of light naphtha ref. [42] became possible. This method has matured over the years towards achieving integrated biorefinery approaches in 2018, reaching the complete valorization of all lignocellulose constituents. For example, value-added products (propanolguaiacol and aliphatic alcohols) were produced in a model biorefinery with complete lignocellulose conversion. Importantly, these were further converted to a plethora of value-added building blocks with focus on amines [64]. The cellulose fraction was fully converted, in supercritical methanol, allowing for catalyst recycling. The aliphatic alcohols obtained in this step were coupled with cyclopentanone and subsequently converted to hydrocarbons, with target of jet-fuel range cyclic alkanes. Furthermore, an elegant liquid phase cellulose-to-naphtha technology was developed [65]. A deep converting ‘lignin-first’ biorefinery concept meaning gasoline and kerosene/diesel production was introduced [66]. Liquid alkylcyclohexanes, arenes, polyols, and furfural derivatives were produced [70,71]. The latest novel results were carried out using new catalysts [80,87,88] and by developing task-specific catalysts for hardwood and softwood [86], chemodivergent hydrogenolysis (for propyl- or propanol-methoxyphenol production) [87], membrane filtration [83], and applying new solvents/stabilization agent (dimethyl carbonate) [92]. The first integrated techno-economic assessment of a biorefinery process revealed that using only waste wood as feedstock can make the investment profitable [101].

In all the RCF systems, where the lignin fraction is valorized ‘first’, the celluloses will remain mixed with the heterogeneous catalyst, which means that the catalyst recycling issue needs to be addressed and many creative solutions have been already found. Possible solutions for catalyst separation were developed using a magnetic catalyst [39], membrane filtration [83], or embedding the metal function in a cage [4]. Adding a second catalytic step that converts all process residues was also developed, which liberated the catalyst for re-use [64].

## 4. Future Perspectives and Challenges

Several achievements have already been reached in ‘lignin-first’ process technology in recent years: noble-metal containing catalysts were replaced by more sustainable metals, or other catalysts; hydrogen was produced self-sufficiently from the pulp or solvent, and a good level of uniformity and chemodivergency of the products using mild conditions and appropriate (task-specific) catalysts has been reached, pointing toward exciting possibilities for ultimately converting total woody biomass into value-added products (as per the ‘no carbon left behind’ strategy). Notably, there were examples for the integration of biorefinery into petrorefinery processes, and the direct usage of lignin oil as a sulphur-free diesel-soluble liquid fuel. Semi-continuous flow-through systems have been established with good efficiency.

Future work will undoubtedly focus on several directions such as replacing batch reactors with really continuous flow-through systems and development of RCF processes applicable to crude biomass and lignocellulosic waste streams (e.g., bark). Effective removal of the catalyst from the pulp to enable subsequent enzymatic or catalytic treatment of the (hemi)cellulose fraction and improving recyclability of the catalysts will be essential to move toward real upscaling efforts, where in addition, the choices of solvent will be very important.

RCF enables to derive more value from lignin by increasing the yield and selectivity of desired aromatic monomers, which enables the more efficient production of well-defined products from lignin, thereby influencing the overall economic feasibility of lignocellulosic biorefineries. In the future, focus will also shift toward establishing downstream processing strategies for all lignocellulose constituents, and the diversification of the product portfolio accessible from lignocellulosic biorefineries.

## Figures and Tables

**Table 1:** ‘Lignin-first’ or reductive catalytic fractionation (RCF) papers in the literature.

Entry	Year	Feedstock	Catalyst	Solvent	Reaction Conditions			Yield of Main Products	Lignin Monomer	Reference
					T (°C)	p (bar)	t(h)	wt %	Yield (wt %)	
1	1940	maple, spruce	Cu_2_Cr_2_O_5_	dioxane	250	333 (H_2_)	12	4-propylcyclohexanol, 4-propylcyclohexanediol		[22]
2	1941	maple, spruce	Cu_2_Cr_2_O_5_	dioxane	280	H_2_	10	4-propylcyclohexanol	36	[23]
3	1943	maple	Cu_2_Cr_2_O_5_	dioxane	280	H_2_	20	4-propylcyclohexanol, 3-cyclohexyl-1-propanol	40	[24]
4	1948	maple	Raney-Ni + NaOH	dioxane/H_2_O (1:1)	173	210 (H_2_)	6	15% 4-ethylsyringol, 6% 4-ethanolsyringol	27	[25]
5	1963	aspen	Raney-Ni	dioxane/H_2_O (1:1)	220	35 (H_2_)	5	29% 4-propylsyringol, 13% 4-propanolsyringol	59	[26]
6	1966	spruce	Pd/C	dioxane/H_2_O (1:1)	195	35 (H_2_)	10	17% 4-propanolguaiacol	17	[27]
7	1969	spruce	Rh/C	dioxane/H_2_O (1:1)	195	35 (H_2_)	5	11% 4-propanolguaiacol	34	[28]
8	1970	spruce	Raney-Ni	dioxane/H_2_O (1:1)	195	35 (H_2_)	5	17% 4-propanol-cyclohexanol	17	[29]
9	1978	aspen	Rh/C	dioxane/H_2_O (1:1)	195	34(H_2_)	5	26% 4-propanolsyringol, 13% 4-propylsyringol	50	[30]
10	1978	spruce	Rh/C	dioxane/H_2_O (1:1)	195	35(H_2_)	5	21% 4-propanolguaiacol	21	[31]
11	1986	aspen poplar	Rh/C	dioxane/H_2_O (1:1)	195	35(H_2_)	5	21% 4-propanolguaiacol	21	[32]
12	1993	rice husks	polyvalent metals	dioxane	250	50(H_2_)	2	33% 4-propylsyringol	33	[33]
13	2008	birch	H_3_PO_4_ + Pt/C	dioxane/H_2_O (1:1)	200	40(H_2_)	4	21% 4-propylsyringol, 15% 4-propanolsyringol	46	[34]
14	2011	pine	Pd/C	dioxane/H_2_O (1:1)	195	35(H_2_)	24	21% 4-propanolguaiacol	22	[35]
15	2012	birch	Ni-W_2_C/C	ethylene glycol	235	60(H_2_)	4	18% 4-propylsyringol, 10% 4-propanolsyringol	47	[36]
16	2013	birch	Ni/C	MeOH	200	1(Ar)	6	36% 4-propylsyringol, 12% 4-propylguaiacol	54	[37]
17	2014	birch	Pd/C	EtOH/H_2_O (1:1)	195	4(Ar)	1	49% 4-propenylsyringol	49	[38]
18	2014	poplar	Raney-Ni	2-PrOH/H_2_O (7:3)	180	autogenous	3	4-propanolsyringol	25	[39]
19	2015	poplar	Zn-Pd/C	MeOH	225	35(H_2_)	12	30% 4-propylsyringol, 24% 4-propylguaiacol	54	[40]
20	2015	birch	Ru/C	MeOH	250	30(H_2_)	6	31% 4-propylsyringol, 10% 4-propylguaiacol	52	[41]
21	2015	lignocellulose	Ru/C + H_4_SiW_12_O_40_	org.phase/H_2_O	140–300	50(H_2_)	< 24	n-hexane, cyclohexane, methylcyclopentane	82 *	[42]
22	2015	corn stalk	Ru/C + LiTaMoO_6_	H_3_PO_4_	230	60(H_2_)	24	6% 4-ethylphenol	24	[43]
23	2015	birch	Pd/C	MeOH	250	30(H_2_)	3	35% propanolsyringol, 10% propanolguaiacol	49	[44]
24	2015	birch	Pd/C	ethylene glycol	250	30(H_2_)	3	35% propanolsyringol, 12% propanolguaiacol	50	[45]
25	2015	birch	Ni/C	MeOH	200	2(N_2_)	6	18% 4-propylsyringol, 10% 4-propylguaiacol	32	[46]
26	2015	cedar	H_2_SO_4_	toluene/MeOH	170	1(air)	0.1	5% homovanillyl aldehyde dimethyl acetal	10	[47]
27	2016	poplar	Ru/C	MeOH	250	40(H_2_)	15	49% propylsyringol, 22% propanolsyringol	78 *	[6]
28	2016	birch	Pd/C	EtOH/H_2_O (1:1)	210	1(Ar)	15	20% 4-propylsyringol, 11% 4-propenylsyringol	36	[48]
29	2016	poplar	Zn-Pd/C	MeOH	225	35(H_2_)	12	28% 4-propylsyringol, 14% 4-propylguaiacol	43	[49]
30	2016	miscanthus	Ni/C	MeOH	225	60(H_2_)	12	19% 4-propylsyringol, 21% 4-propylguaiacol	68	[50]
31	2016	poplar	(Rh(cod)Cl)_2_ + Sc(OTf)_3_	Dioxane/H_2_O	175		2	3% 4-propenylsyringol, 2% 4-methylguaiacol	10	[51]
32	2016	birch	Pd/C + Yb(OTf)_3_	MeOH	200	30(H_2_)	2	4-propanolsyringol, 4-propanolguaiacol	48	[52]
33	2016	beech	Ni/C	MeOH/H_2_O (3:2)	200	60(H_2_)	5	29% 4-propanolsyringol, 10% 4-propanolguaiacol	51	[53]
34	2016	corn stover	H_3_PO_4_ + Ni/C	MeOH	200	30(H_2_)	6	15% methyl coumarate, 15% methyl ferulate	38	[54]
35	2016	poplar	H_3_PO_4_ + Pd/C	MeOH	200	20(H_2_)	3	21% 4-propanolsyringol, 14% 4-propanolguaiacol	42	[55]
36	2016	poplar	Pd/C	MeOH/H_2_O (7:3)	200	20(H_2_)	3	23% 4-propanolsyringol, 13% 4-propanolguaiacol	44	[56]
37	2017	birch	Pd/C + Al(OTf)_3_	MeOH	180	30(H_2_)	2	34% methoxypropylsyringol, 8% methoxypropylguaiacol	55	[57]
38	2017	oak	Pd/C + Al(OTf)_3_	MeOH	180	30(H_2_)	2	12% methoxypropylsyringol, 10% propanolsyringol	46	[58]
39	2017	birch	NiFe/C	MeOH	200	20(H_2_)	6	24% 4-propylsyringol, 11% 4-propylguaiacol	40	[59]
40	2017	*pinus radiata*	Pd/C	Dioxane/H_2_O (1:1)	195	34(H_2_)	24	22% propanolguaiacol, 3% propylguaiacol	78 ^+^	[60]
41	2017	birch	Ni/Al_2_O_3_	MeOH	250	30(H_2_)	3	21% propanolsyringol, 5% propylsyringol	36	[4]
42	2017	birch	H_3_PO_4_ + Pd/C	MeOH/H_2_O (7:3)	180	30(H_2_)	3	18% propanolsyringol, 11% propylsyringol	37	[61]
43	2017	poplar	Ni/C	MeOH	190	60(H_2_)	3	12% propylguaiacol and propylsyringol	17	[62]
44	2018	poplar	Ni/C	MeOH	200	30(H_2_)	1	8% propylsyringol, 5% propylguaiacol	48	[63]
45	2018	pine	Cu_20_PMO	MeOH	220	40(H_2_)	18	8% propanolguaiacol, 4% propylguaiacol	13	[64]
46	2018	birch	Ru/C + H_4_SiW_12_O_40_	petrol/H_2_O	220	50(H_2_)	5	21% propanolsyringol, 5% propylsyringol	39	[65]
47	2018	poplar, spruce	Raney-Ni, Ni_2_P/SiO_2_	2-PrOH/H_2_O (7:3)	180	autogenous	3	50–60% phenolic species	20–25	[66]
48	2018	poplar, spruce	Raney-Ni	2-PrOH/H_2_O (7:3)	200	autogenous	6			[67]
49	2018	sorghum	Fenton	EtOH	250	autogenous	12	phenolic oil	76	[68]
50	2018	oak	Al(OTf)_3_ + Pd/C	MeOH	160	autogenous	2	9% propylsyringol, 5% propylguaiacol	25	[69]
51	2018	cornstalk	Ru/C	H_2_O	200	30(H_2_)	8	67% ethylcyclohexane, 16% propylcyclohexane	97 *	[70]
52	2018	birch	Pd/C+Yb(OTf)_3_	MeOH	250	20(H_2_)	20	lignin-oil	83 *	[71]
53	2018	eucalyptus	Ru/C	BuOH/H_2_O (1:1)	200	30(H_2_)	2	41% propanolsyringol and propanolguaiacol	49	[72]
54	2018	bark	Pd/C	MeOH/H_2_O (2:1)	200		2	3% ethylguaiacol	42	[73]
55	2018	vanilla seeds	Ni/C	MeOH	250	30(H_2_)	3	18% propylcatechol, 3% propenylcatechol	21	[74]
56	2018	corncob	ZnMoO_4_/MCM-41	MeOH	220	30(H_2_)	4	16% methyl coumarate, 13% methyl ferulate	38	[75]
57	2018	cork	Rh/C	2-methyl tetrahydrofuran	200	40(H_2_)	4	bio-oil	43	[76]
58	2018	apple wood	Ru/SiC	MeOH	250	10(H_2_)	3	propylsyringol and ethylsyringol	48	[77]
59	2018	corn stover	MoS_2_	MeOH	20	30(H_2_)	2	4% propylguaiacol, 3% ethylphenol	18	[78]
60	2018	birch	CdS	MeOH/H_2_O	r.t.	1(N_2_)	8	14% propanonesyringol, 7% propanoneguaiacol	27	[79]
61	2019	birch	Co-phen/C	EtOH/H_2_O (1:1)	200	autogenous	4	10% 4-propylsyringol, 9% 4-propenylsyringol	34	[80]
62	2019	oak	Ni-Al/AC	HCOOH/EtOH/H_2_O	190	autogenous	3	9% propylsyringol, 5% propylguaiacol	23 ^x^	[81]
63	2019	birch	Pt/Al_2_O_3_	MeOH/H_2_O (1:2)	230	autogenous	3	40% propylsyringol, 6% propylguaiacol	49	[82]
64	2019	poplar	Raney-Ni	2-PrOH/H_2_O (7:3)	200	autogenous	3	11% propanolsyringol, 10% propanolguaiacol	34	[83]
65	2019	poplar, spruce	Raney-Ni	2-PrOH/H_2_O (7:3)	220	autogenous	3	10% ethylsyringol, 7% propylsyringol	36	[84]
66	2019	poplar	Ni/C	MeOH	225	35(H_2_)	12	propylsyringol, propylguaiacol	90	[85]
67	2019	apple wood	Mo_x_C/CNT	MeOH	250	10(H_2_)	3	propylsyringol, propylguaiacol	42	[86]
68	2019	eucalyptus	Ni@ZIF-8	MeOH	260	30(H_2_)	8	24% propylsyringol + propylguaiacol	44	[87]
69	2019	basswood	binuclear Rh complex	NaOH/H_2_O	110	1(Ar)	24	2% propanonesyringol, 1.6% ethanonesyringol	5	[88]
70	2020	poplar	binuclear Rh complex	NaOH/H_2_O	110	1(Ar)	12	9% ethanonesyringol, 6% ethanoneguaiacol	17	[89]
71	2020	birch	Ru/C	MeOH	235	30(H_2_)	3	20% phenol, 9% propylene	29	[90]
72	2020	eucalyptus sawdust	Ru/C	BuOH/H_2_O (1:1)	200	30(H_2_)	2	propanol-substituted phenolics	49	[91]
73	2020	pine	H_2_SO_4_	dimethyl carbonate	140	autogenous	0.67	8% G-C2-acetal	9	[92]
74	2020	eucalyptus	Pd/C	MeOH	240	30(H_2_)	4	32% propanolsyringol, 13% propanolguaiacol	50	[93]
75	2020	beech	NiMo/Al_2_O_3_	EtOH	260	26(H_2_)	3	11% propylsyringol, 6% propylguaiacol	20	[94]
76	2020	pine	Pt/C	MeOH/H_2_O	230	30(H_2_)		10 mol% phenol	15	[95]
77	2020	birch	H-BEA	EtOH/H_2_O	220		2		20	[96]
78	2020	poplar			250	autogenous	0.33			[97]
79	2020	birch		H_2_O	195					[98]
80	2020	poplar	emimAce	emimAce	110	1(air)	4	38% lignin		[99]
81	2020	maple	Zr-KIT-5	γ-valerolactone	250	30(H_2_)	18	3.5% 2-phenylpropan-2yl acetate, 1.6% 3,4-dimethoxyphenol	7	[100]

* mol%, ^+^ oil yield in wt %, ^x^ C%.
